# Nuclear Spin‐Free ^70^Ge/^28^Si^70^Ge Quantum Well Heterostructures Grown on Industrial SiGe‐Buffered Wafers

**DOI:** 10.1002/advs.202523504

**Published:** 2026-02-16

**Authors:** Patrick Daoust, Nicolas Rotaru, Debojyoti Biswas, Sebastian Koelling, Eloise Rahier, Alexis Dubé‐Valade, Patrick Del Vecchio, Marcus S. Edwards, Mukhlasur Tanvir, Ebrahim Sajadi, Joe Salfi, Oussama Moutanabbir

**Affiliations:** ^1^ Lassonde DeepTech Institute, Department of Engineering Physics École Polytechnique de Montréal Montréal Québec Canada; ^2^ Stewart Blusson Quantum Matter Institute University of British Columbia Vancouver BC Canada; ^3^ Department of Electrical and Computer Engineering University of British Columbia Vancouver BC Canada; ^4^ Department of Physics and Astronomy University of British Columbia Vancouver BC Canada

**Keywords:** isotopically enriched semiconductors, Ge/SiGe heterostructures, spin qubit, nuclear spin, epitaxy, atom probe tomography, magneto‐transport

## Abstract

The coherence of hole spin qubits in germanium planar heterostructures is limited by the hyperfine coupling to the nuclear spin bath due to 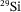 and 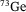 isotopes. Thus, removing these nuclear spin‐full isotopes is essential to extend the hyperfine‐limited coherence times needed to implement robust quantum processors. This work demonstrates the epitaxial growth of device‐grade nuclear spin‐free 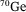/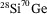 heterostructures on industrial SiGe buffers while minimizing the amounts of highly purified 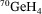 and 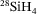 used. The obtained 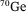/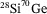 heterostructures exhibit a dislocation density of $5.3\times 10^{6}\;{\mathrm{cm}}^{-2}$ and an isotopic purity exceeding 99.99%, with carbon and oxygen impurities below the detection sensitivity, as revealed by atom probe tomography. Magneto‐transport measurements on gated Hall bars demonstrate effective gate control of hole density in nuclear spin‐free quantum wells. Negative threshold gate voltages confirm the absence of intentional doping in the wells, while Hall and Shubnikov–de Haas analyses yield consistent carrier densities ($∼1.4\times 10^{11}\;{\mathrm{cm}}^{-2}$) and high mobilities ($∼2.4\times 10^{5}\;{\mathrm{cm}}^{2}/\mathrm{Vs}$). Mobility trends reveal interface‐trap‐limited scattering and percolation concentration below $7\times 10^{10}\;{\mathrm{cm}}^{-2}$. These analyses, along with atomic‐level studies, confirm the high quality of epitaxial 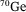/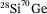 heterostructures and their relevance as a platform for long‐coherence spin qubits.

## Graphical abstract

Hyperfine coupling to  and  nuclear spins limits hole spin‐qubit coherence in Ge heterostructures. We demonstrate device‐grade, nuclear‐spin‐free / quantum wells grown on industrial SiGe buffers with minimal use of enriched precursors. Atom‐probe and magnetotransport measurements reveal exceptional isotopic purity, low defect density, and high mobilities, establishing a robust platform for long‐coherence spin qubits.

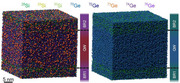

## 1 Introduction

Germanium (Ge) and its alloys have emerged as a promising platform to implement scalable quantum processors, leveraging robust Ge‐based hole spin qubits [1, 2, 3, 4, 5, 6, 7, 8, 9, 10, 11, 12, 13, 14, 15, 16, 17, 18, 19, 20]. Besides its compatibility with silicon processing standards, Ge offers a quiet quantum environment without valley degeneracy suitable to engineer hole spin states within scalable device structures with favorable properties for qubit control and coherence. The large spin–orbit interaction (SOI) in Ge enables all‐electrical manipulation of spin states, i.e., control of spin via the charge degree of freedom, eliminating the need for oscillating magnetic fields and allowing faster and more scalable qubit control [21]. The pronounced SOI provides tunable g‐factors through electric fields, offering versatile pathways to optimize spin coherence and coupling in quantum dot architectures [1]. Moreover, it is generally expected that holes experience a weaker hyperfine interaction than electrons owing to the predominantly p‐type symmetry of their wavefunctions, which vanishes at the nucleus. This property initially provided an additional motivation to pursue the development of hole‐spin qubits. However, theoretical studies have shown that the hyperfine coupling for holes can be only an order of magnitude smaller than that of electrons [22, 23], and in some cases comparable to that in silicon [23]. Furthermore, the p‐orbital character combined with d‐orbital hybridization gives rise to pronounced anisotropy in the hole hyperfine interaction, a feature absent in electron spins [22].

The extent of the hyperfine interactions was recently addressed in a study probing both charge noise and hyperfine‐mediated magnetic noise in hole spin qubits embedded in planar strained Ge/${\mathrm{Si}}_{x}{\mathrm{Ge}}_{1-x}$ heterostructures [24]. That work, based on echo envelope modulations and noise‐spectrum fitting, revealed that residual coupling to 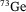 and 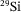 nuclear spin bath contributes significantly to dephasing, setting an approximate $T_{2}^{*}$ bound of ∼1μs under their natural isotopic composition. The study further estimates that fully isotopically purified Ge and surrounding ${\mathrm{Si}}_{1-x}{\mathrm{Ge}}_{x}$ barriers could push the hyperfine‐limited coherence times into the tens to hundreds of microseconds regime. Furthermore, a recent experimental study demonstrated that, due to the anisotropy of the hyperfine coupling and the g‐tensor, in the presence of inhomogeneous strain, out‐of‐plane magnetic fields are optimal for mitigating decoherence induced by electric field fluctuations, whereas in‐plane magnetic fields are more effective in suppressing decoherence arising from the nuclear spin bath [25]. These studies show that removing nuclear spins is a critical pathway to improve coherence for hole spin qubits. These observations confirm that developing nuclear spin‐free Ge heterostructures is essential toward practical hole spin qubits.

**FIGURE 1:**
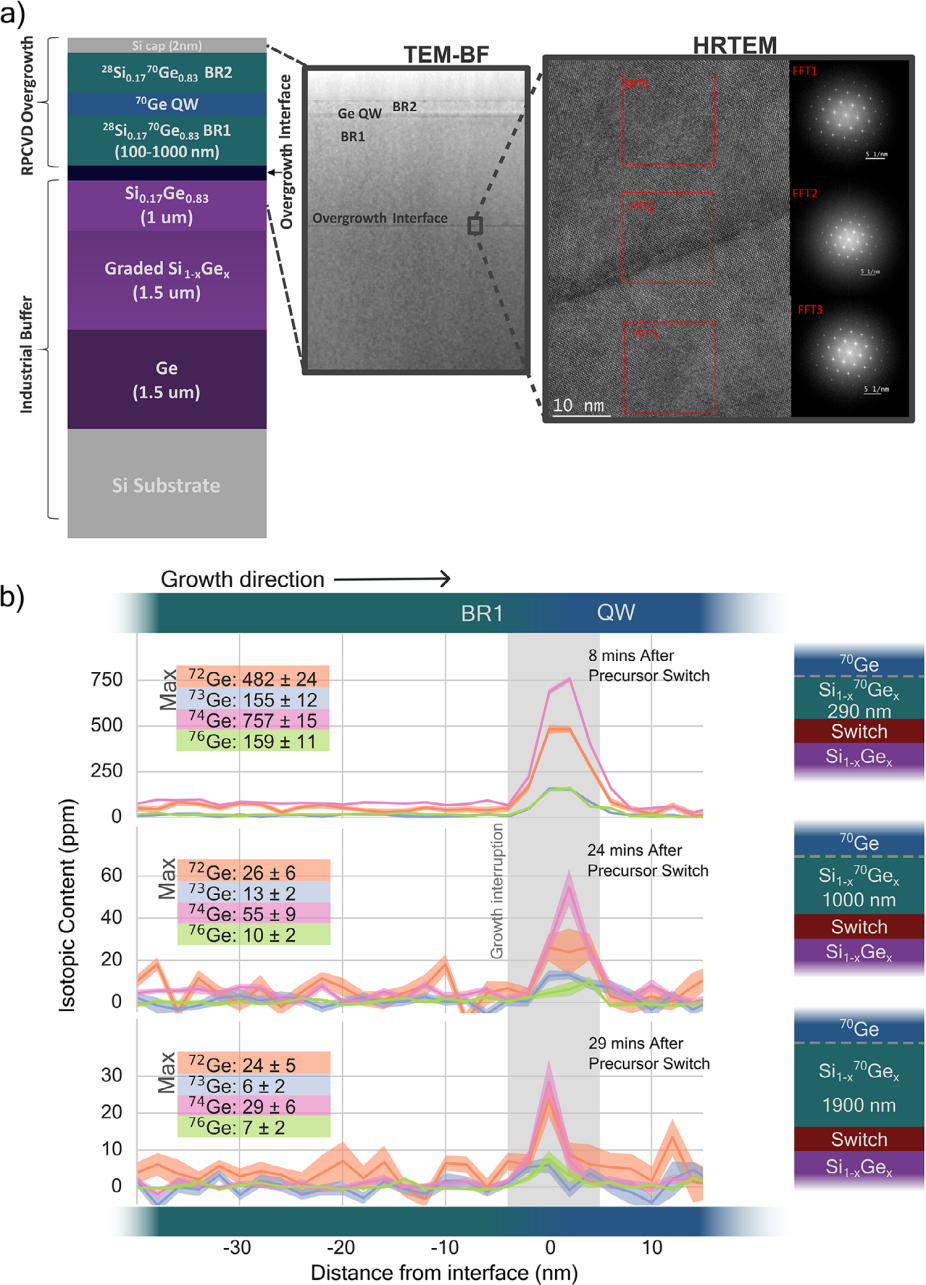
(a) Schematic illustration of the growth stack used to grow Ge/SiGe heterostructures on industrial SiGe buffers. Inset 1: Low‐magnification TEM image of a typical 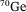/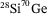 heterostructure. Inset 2: High‐resolution TEM micrograph recorded at the regrowth interface. (b) Atom probe tomography (APT) profiles of Ge isotopes in Ge/SiGe heterostructures obtained under different growth protocols. The vertical shadowed band approximately marks the transition region following the growth pause as established from the reservoir effect‐induced change in composition measured by APT.

Recent attempts to grow isotopically controlled 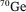/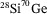 heterostructures [26], using purified 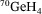 and 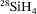 precursors, demonstrate that cross‐contamination from natural precursors in the growth reactor makes the complete removal of nuclear spin‐full nuclei 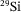 and 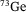 rather difficult and somewhat impractical. The growth of planar Ge/SiGe heterostructures on silicon typically involves the initial growth of a thick strained‐relaxed ${\mathrm{Si}}_{1-x}{\mathrm{Ge}}_{x}$ buffer layer, ideally using conventional precursors with natural isotopic content (e.g., 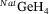 and 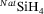). The heterostructure, consisting of barriers and a quantum well (QW), is subsequently grown by switching to isotopically purified precursors. However, traces of natural precursors in the reactor can hardly be eliminated during this last step, leading to the undesirable incorporation of nuclear spin‐full species in the grown 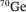/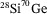 heterostructures [26]. Although this reservoir effect can be greatly suppressed by using exclusively 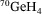 and 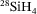 to grow the entire several‐micrometer‐thick stack (e.g., Figure 1a), including the relaxed ${\mathrm{Si}}_{1-x}{\mathrm{Ge}}_{x}$ buffers, this approach remains infeasible because it requires excessive amounts of rare and costly isotopically enriched precursors.

Herein, we circumvent these challenges by establishing protocols for the epitaxial growth of nuclear spin‐free 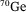/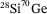 heterostructures directly on 200 mm industrial ${\mathrm{Si}}_{x}{\mathrm{Ge}}_{1-x}$‐buffered silicon wafers, as illustrated in Figure 1a. The use of ex situ‐grown SiGe layers effectively suppresses cross‐contamination and significantly reduces the consumption of isotopically enriched precursor gases, without compromising the crystalline quality. This approach enables the realization of nuclear‐spin‐free heterostructures with no traces of carbon and oxygen impurities. Moreover, Hall measurements reveal precise electrostatic gate control of the hole density and high mobilities, with transport predominantly limited by interface trap scattering. Collectively, these results establish high‐purity 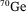/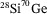 heterostructures as a compelling platform for long‐coherence hole spin qubits, intrinsically free from hyperfine interactions associated with 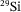 and 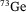 nuclei.

## 2 Experimental

A meticulous surface‐cleaning process was developed to properly condition the ${\mathrm{Si}}_{x}{\mathrm{Ge}}_{1-x}$ surface to enable the growth of 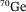/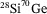 heterostructures employing a mix of wet chemical treatment and in situ annealing, which we subsequently describe in detail. Note that the cleaning processes previously developed for Ge [27] and ${\mathrm{Si}}_{x}{\mathrm{Ge}}_{1-x}$ [28] surfaces are not effective for Ge‐rich ${\mathrm{Si}}_{x}{\mathrm{Ge}}_{1-x}$ surfaces investigated here. The quality of the heterostructures was assessed through detailed investigations of their microstructure and atomic‐level 3D isotopic composition using transmission electron microscopy (TEM), X‐ray diffraction (XRD), atomic force microscopy (AFM), and atom probe tomography (APT). Magneto‐transport measurements further confirm the high crystallinity and excellent interfacial quality of the obtained nuclear spin‐free 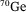/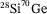 heterostructures.

The growth was carried out on the pre‐conditioned ${\mathrm{Si}}_{x}{\mathrm{Ge}}_{1-x}$ surfaces in a reduced‐pressure chemical vapor deposition reactor equipped with isotopically purified 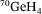 (isotopic purity >99.9%) and 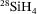 (isotopic purity >99.99%) precursors. These precursors contain only traces of other Si (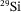 and 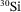) and Ge (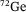, 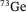, 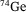, and 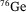) isotopes, with a combined total content below 0.006at.% in each precursor. The presence of chemical contaminants, including residual hydrides, is also negligible, with an average content of less than 0.06μmol/mol. After systematic studies (see Methods), 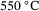 is identified as the optimal growth temperature that yields high‐quality growth and sharp interfaces. To assess the ability of this process to eliminate cross‐contamination, reference samples were also investigated. In these samples, both the heterostructure and the buffer layer were grown in the same reactor by switching from natural to purified precursors [26].

## 3 Results and Discussion

Figure 1b exhibits the APT profiles of the isotopic content along the growth direction from the ${\mathrm{Si}}_{1-x}{\mathrm{Ge}}_{x}$ buffer layer up to the 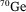 quantum well (QW) for the reference samples for which the switch to purified precursor took place at different depths from the QW interface (i.e., different times from the QW growth). For simplicity, only the profiles of Ge isotopes are displayed. Note that a 90 s growth pause was introduced just before the QW growth to ensure a sharp ${\mathrm{Si}}_{x}{\mathrm{Ge}}_{1-x}$/Ge interface. The APT profiles clearly reveal the incorporation of undesired Ge isotopes within the QW. Even after growing a 1.9 μm‐thick ${\mathrm{Si}}_{1-x}{\mathrm{Ge}}_{x}$ layer using purified precursors, corresponding to 29 min after halting the supply of natural precursors, a measurable isotopic carry‐over remains (bottom profile). As expected, this reservoir effect becomes more pronounced when the growth time after precursor switching is shorter. For example, only 8 min after switching (top profile), the maximum concentration of 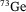 in the QW exceeds 150 ppm.

Eliminating this cross‐contamination can be achieved by establishing the growth of 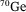/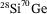 heterostructures on ${\mathrm{Si}}_{x}{\mathrm{Ge}}_{1-x}$ wafers (Figure 1a). Surface conditioning prior to growth is the main challenge in growing high‐quality heterostructures in *ex situ* grown ${\mathrm{Si}}_{x}{\mathrm{Ge}}_{1-x}$ buffers. In general, cleaning ${\mathrm{Si}}_{x}{\mathrm{Ge}}_{1-x}$ surfaces prior to epitaxy can be a complicated and daunting undertaking due to the complex nature of Si and Ge native oxides and related surface chemistry [27, 28]. The mixed ${\mathrm{SiO}}_{2}$–${\mathrm{GeO}}_{2}$ oxide exhibits non‐uniform thermal desorption behavior: while ${\mathrm{GeO}}_{2}$ is volatile and decomposes around 400–600 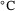, ${\mathrm{SiO}}_{2}$ remains stable at higher temperatures. Moreover, ${\mathrm{GeO}}_{2}$ can react with Si to form ${\mathrm{SiO}}_{2}$ and elemental Ge, leading to Ge enrichment and surface roughening during annealing. Ge also segregates to the surface under thermal or chemical treatment, promoting reoxidation and non‐uniform hydrogen termination after HF‐based cleans. These effects, compounded by a narrow thermal window for oxide desorption, make atomically clean, ordered ${\mathrm{Si}}_{x}{\mathrm{Ge}}_{1-x}$ surfaces difficult to achieve as compared to pure Si or Ge.

Here, a multi‐step cleaning process involving diluted HF and HCl cleans, as well as in situ hydrogen anneals at high temperature, is performed before the epitaxial growth of the 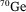/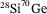 heterostructure. The optimized surface preparation protocol is summarized in the Methods section and in Table S1 (Supporting Information).

The epitaxial growth on ${\mathrm{Si}}_{x}{\mathrm{Ge}}_{1-x}$ buffers starts with an initial boost of 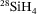 to prevent the formation of Ge 3D islands. Once the growth of the first barrier (BR1) is completed, the precursor flows were cut for approximately 30 s, during which the reactor is purged with 2700 sccm of hydrogen flow. The growth of the 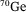 quantum well (QW) then follows with a subsequent 30 s hydrogen purge of the reactor. To ensure a sharp interface between the well and the second barrier (BR2), the 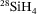 precursor flow is momentarily increased through a second boost step. Finally, a ∼2 nm capping layer of 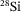 is grown to protect the purified heterostructure with a stable native oxide. The heterostructure dimensions were optimized following systematic growth experiments to obtain a uniform thickness for each layer within a range ensuring heavy‐hole confinement while keeping its wavefunction away from the noisy surface, as confirmed by k·p theory calculations (not shown).

Figure 1a (inset) displays a low‐magnification TEM image of a representative heterostructure, confirming that the growth protocol described above yields heterostructures free of extended defects (at the TEM scale) with a uniform thickness. Note that the homoepitaxial layer grown directly on the strain‐relaxed ${\mathrm{Si}}_{x}{\mathrm{Ge}}_{1-x}$ buffer is also defect‐free. Indeed, high‐resolution TEM imaging and related diffraction patterns at the regrowth interface demonstrate that the grown layers display the same lattice structure as that of the industrial buffers, as exemplified in Figure 1a (inset). The noticeable change in contrast at the interface is attributed to the formation of a Ge‐rich layer at the onset of growth, as confirmed by APT (Supporting Information, Section 4).

**FIGURE 2:**
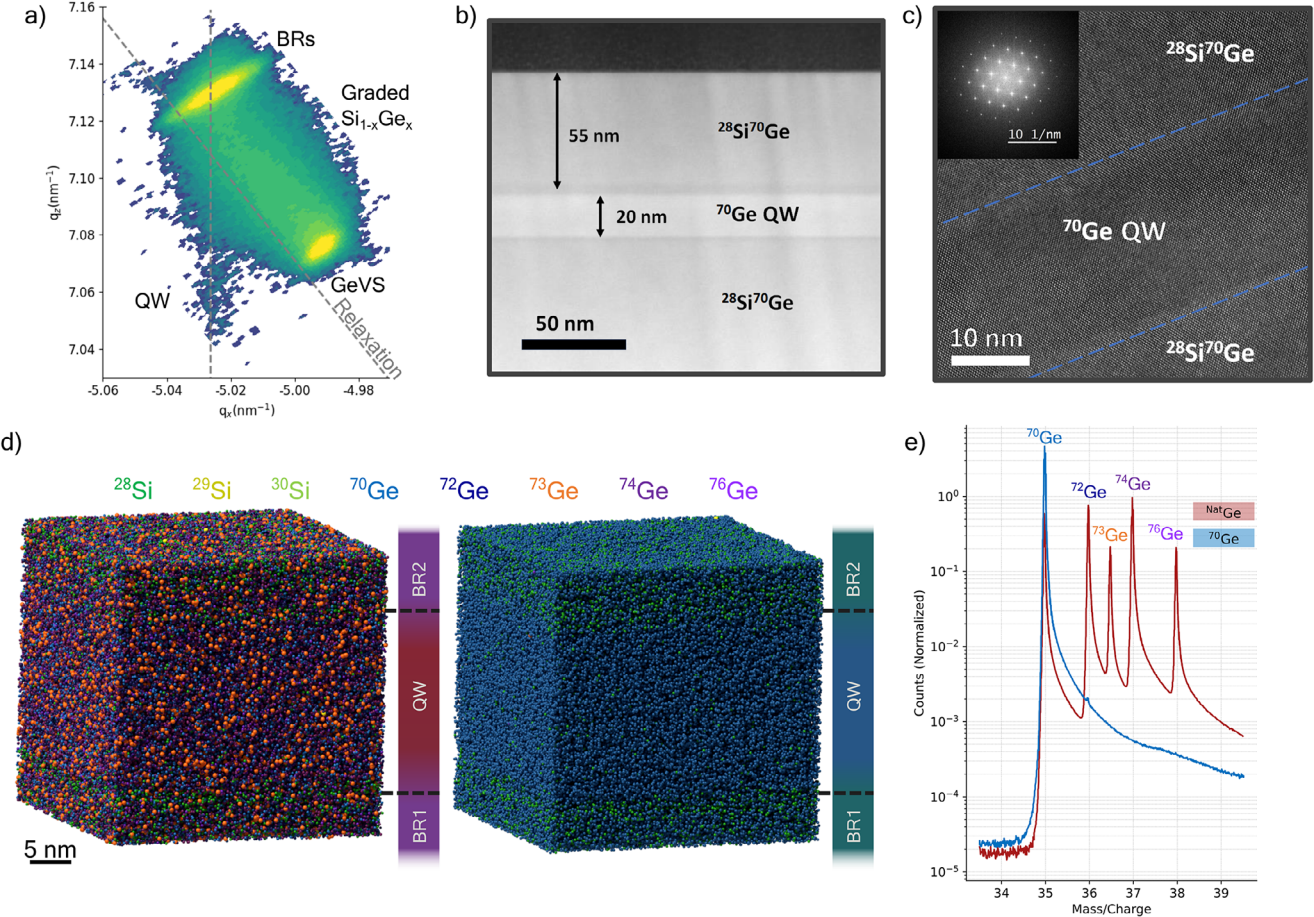
(a) XRD‐RSM recorded around the ($\bar{2}\;\bar{2}\;4$) reflection for an optimized 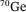/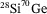 heterostructure showing the relaxation line (diagonal dashed line) and quantum well pseudomorphism line (vertical dashed line). (b) Annular dark‐field TEM image of an as‐grown 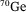/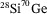 heterostructure with a 20 nm‐thick quantum well and a 55 nm‐thick top barrier. (c) High‐resolution TEM image of the 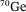 quantum well and its interfaces with the SiGe barriers. (d) 3D isotope‐by‐isotope maps of QW structures grown with natural precursors (left) and isotopically purified precursors (right). (e) APT mass spectra showing the detected Ge isotopes for the heterostructures in (d).

Further insights into the basic structural properties of the grown heterostructures are obtained from high‐resolution XRD reciprocal space mapping (XRD‐RSM) analyses. Figure 2a displays a typical XRD‐RSM recorded around the ($\bar{2}\;\bar{2}\;4$) reflection for an optimized heterostructure with a 20 nm‐thick Ge QW and a 55 nm‐thick ${\mathrm{Si}}_{x}{\mathrm{Ge}}_{1-x}$ BR2. Figure 2b,c shows annular dark‐field TEM and high‐resolution TEM images of this heterostructure, respectively. Although the quantum well is only 20 nm in thickness, its signature can be clearly observed in the XRD‐RSM under the sharp overlapping reflections of the ${\mathrm{Si}}_{x}{\mathrm{Ge}}_{1-x}$ (x=0.17) buffer and barriers. As there is no deviation from the pseudomorphism line (vertical dashed line), the grown QW did not relax and thus remains fully compressively strained. The composition of the barriers was determined using a Vegard‐type model [29], yielding 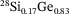, identical to that of the underlying relaxed buffers.

The roughness of as‐grown heterostructures was investigated using AFM (SI). Prior to growth, the industrial buffers exhibit an average and a root‐mean‐square roughness of $R_{a}=1.5$ nm and $R_{q}=2.0$ nm, respectively. Surface conditioning slightly increases these values to $R_{a}=2.1$ nm and $R_{q}=2.7$ nm, which remain unchanged after the 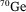/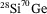 heterostructure growth. This behavior is consistently observed regardless of the thickness of the first barrier (BR1), which was varied between 100 nm and 1 μm. Standard Secco etch [30] defect delineation studies were subsequently performed to probe the dislocation density in the grown materials. Etch pits, attributed to threading dislocations, were found to show densities of $7.8\times 10^{6}\;{\mathrm{cm}}^{-2}$ and $5.3\times 10^{6}\;{\mathrm{cm}}^{-2}$ for the industrial buffers and grown heterostructures, respectively. These dislocation densities are consistent with the literature [31] and confirm the high quality of the grown heterostructures. More details on these studies are provided in Section S3 (Supporting Information).

To elucidate the isotopic and chemical purity of the as‐grown heterostructures, laser‐assisted atom probe tomography (APT) measurements were carried out using a picosecond laser with a wavelength of 257 nm and a pulse energy of 25–45 pJ. The analyses were performed at a base temperature of 25 K. Figure 2d displays representative APT 3D isotope‐by‐isotope reconstructed maps of a typical natural Ge/SiGe heterostructure (left) and an optimized isotopically controlled 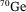/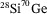 heterostructure (right). In the natural heterostructure, nuclear spin‐full isotopes 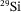 and 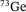 are clearly visible in both the quantum well (QW) and the barrier layers. The average distance between nuclear spins in this heterostructure is 0.3–0.4 nm. In contrast, 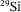 and 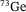 are not detected in the map of the 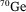/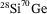 heterostructure. The Ge mass‐to‐charge spectra corresponding to these maps are shown in Figure 2e. Note that in the natural Ge/SiGe heterostructure, five peaks associated with naturally occurring Ge isotopes are observed, whereas only a single peak is detected for 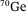/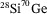, confirming the isotopic purity of these heterostructures.

**FIGURE 3:**
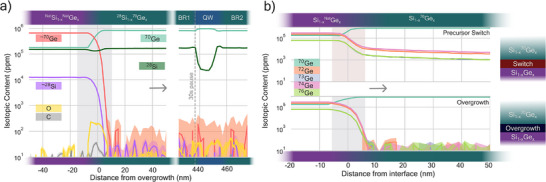
(a) APT compositional profiles near the overgrowth interface showing carbon (C) and oxygen (O) impurities as well as Si and Ge isotopes other than 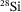 and 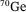, labeled as 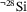 and 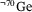. (b) APT profiles of Ge isotopes in heterostructures grown on SiGe‐buffered silicon (bottom) and in a heterostructure grown by switching from natural to purified precursors (top). Note that the content axis is plotted on a logarithmic scale. The arrows indicate the growth direction.

Figure 3a displays the composition profiles of 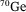 and 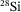 along the growth direction from about 100 nm underneath the growth interface up to the surface. The profiles of C and O impurities are also displayed. The 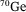 and 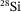 contents in the heterostructure are 83% and 17%, respectively, and remain stable throughout the barrier thickness. These values are consistent with the XRD‐RSM data described above. Furthermore, the composition of the alloy stabilizes within about 30 nm beyond the interface, suggesting that the thickness of BR1 could be further reduced below 100 nm. Apart from a peak of O with a content below 300 ppm at the growth interface, attributed to residual oxide from surface preparation, the levels of O and C impurities in the heterostructure are too close to the APT sensitivity limit, established to be 10 ppm for the current binning of 2 nm, to be meaningful. The concentrations of isotopes other than 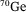 and 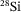, labeled as 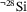 and 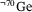, drop rapidly within a few nanometers of the regrowth interface to reach 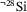 = 11 ppm ± 3 ppm and 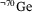 = 41 ppm ± 8 ppm on average over the first 200 nm beyond the interface. This corresponds to an isotopic purity, defined as the ratio of the content of an isotope to the total element content, higher than 99.99%. This rules out any diffusion of material from the buffers or cross‐contamination from past growth experiments. This is further evidenced in Figure 3b, which compares the profiles of Ge isotopes for a heterostructure overgrown on a SiGe‐buffered wafer (Overgrowth) to that of a heterostructure grown by switching the precursors from natural to isotopically enriched sources during the growth of the buffers (Precursor Switch). In particular, in the former, there is no tail associated with the reservoir effect or residues at the growth front, as observed when the buffers are grown in the same reactor using natural precursors.

**FIGURE 4:**
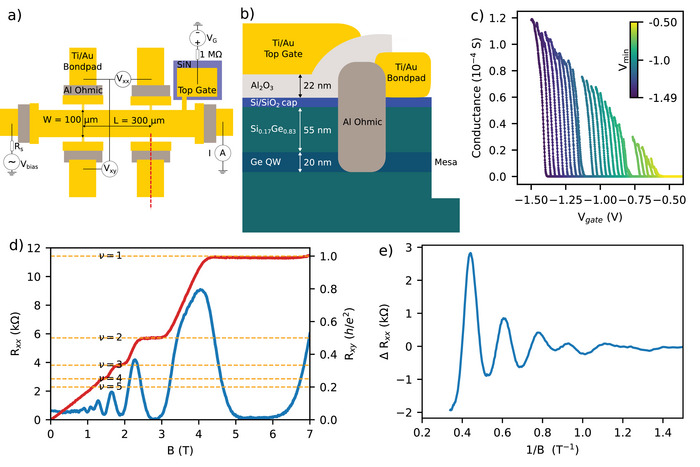
(a) Measurement setup and schematic of a gated Hall bar (HB), showing the excitation voltage $V_{\mathrm{bias}}$ and measured quantities $V_{xx}$ and $V_{xy}$, with $R_{s}=10\;M\Omega$ and $V_{\mathrm{bias}}=40\;\mathrm{mV}$ for magnetotransport measurements, and measured current I with $R_{s}=0\;\Omega$ and $V_{\mathrm{bias}}=50\;\mu V$ for two‐point measurements. The red dashed line indicates the cross‐sectional area through the device shown in (b). (b) Cross‐section of the Ge/SiGe heterostructure and gated Hall bar, including the alloyed Al ohmic contact formed without a via to the quantum well, ohmic bond pad, deposited gate dielectric, and top gate. (c) Two‐point measurement showing turn‐on conductance curves at 1.38 K for a representative HB device, recorded for a range of $V_{\min}$ sweep values from −0.5 V (yellow) to −1.5 V (blue). (d) Magnetotransport up to $B_{⊥}=7$ T at 1.38 K and $V_{\mathrm{gate}}=-2.55$ V, showing $R_{xy}$ (red curve) and $R_{xx}$ (blue curve). The orange dashed lines indicate the integer Landau level filling factors. (e) The corresponding Shubnikov–de Haas (SdH) oscillations are extracted by subtracting an even polynomial background from the data in (d).

The analyses above demonstrate that the introduced growth protocol yields 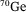/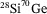 heterostructures of high crystalline quality and high chemical and isotopic purities, thus laying the foundation for transport studies. To this end, gated six‐terminal Hall bars (HBs) were fabricated using optical lithography on the purified heterostructures. Figure 4a,b illustrates the device layout and its cross‐section, respectively. The devices investigated here have a length L=300μm between the voltage contacts and a width W=100μm. The HBs were measured in a cryo‐free variable temperature insert (VTI) at a temperature of approximately 1.4 K, in the bore of an 8 T superconducting solenoid.

For magneto‐transport measurements, we measured the longitudinal $R_{xx}=V_{xx}/I$ and transversal $R_{xy}=V_{xy}/I$ resistances by applying an oscillating bias voltage $V_{\mathrm{bias}}=40\;\mathrm{mV}$ to a resistor $R_{s}=4\;M\Omega \gg R_{xx}$ in series with the device, resulting in a current $I=V_{\mathrm{bias}}/R_{s}=10\;\mathrm{nA}$. Gate voltage $V_{G}$ and temperature were kept constant during sweeps of the perpendicular magnetic field $B_{⊥}$. The gate voltage $V_{G}$ was swept from 0 to $V_{\min}$, then from $V_{\min}$ back to zero, for values of $V_{\min}$ starting at zero and becoming increasingly negative. This procedure was performed to study the effect of gate‐induced charging of interface traps, which shift the threshold voltage. Measurements were repeated by releasing these trapped charges and restoring the unshifted turn‐on voltage by cycling the VTI temperature to 300 K and back to 1.4 K. After a single thermal cycle to reset the device after initial investigations, all data presented in the manuscript were taken sequentially without further cycling.

For the HB measurements, we first verified the ability of a voltage applied to the gate of the HB to control the conductance and hole concentration in the quantum well (QW). Measurements are shown in Figure 4c for a representative gated HB, as a function of $V_{G}$, for several values of $V_{\min}$. When $V_{G}$ is swept from 0 to $V_{\min}<0$, and back to 0, all devices in the fabrication run turned on at a gate voltage of approximately −0.6 V. Forward and backward sweeps for specific $V_{\min}$ values exhibited a hysteresis, confirming the presence of charge trapping, and as $V_{\min}$ is decreased, the turn‐on $V_{G}$ shifts to more negative values, confirming an overall increase in positively trapped interface charges. This has previously been reported in natural Ge/${\mathrm{Si}}_{x}{\mathrm{Ge}}_{1-x}$ heterostructures [32]. The threshold voltage for turn‐on being negative verifies that the isotopically purified QWs are free of carriers at zero gate voltage, i.e., there is no unintentional doping.

For simplicity, we first focus on magneto‐transport measurements at a fixed gate voltage $V_{G}$ and carrier concentration p. The data shown in Figure 4d exhibit, at very low magnetic fields, the Hall effect, i.e., the transverse resistance $R_{xy}$ is proportional to $B_{⊥}$. From the expression for the Hall resistance $R_{xy}=V_{xy}/I_{xx}=B_{⊥}/\left(ep\right)$, where e is the elementary charge, we extract a hole concentration $p=1.49\times 10^{11}\;{\mathrm{cm}}^{-2}$. Increasing the magnetic field $B_{⊥}$ further, we observe Shubnikov–de Haas (SdH) oscillations, periodic in $1/B_{⊥}$, as Landau levels pass through the Fermi energy and modulate the longitudinal resistance. For magnetic fields around 2.7 T and higher, when Landau levels pass through the Fermi energy, we observe the integer quantum Hall effect, with well‐defined transverse resistance $R_{xy}$ plateaus and zero longitudinal resistance $R_{xx}$ for the ν=2 and ν=1 Landau levels at $p=1.49\times 10^{11}\;{\mathrm{cm}}^{-2}$ and T=1.38 K. The SdH oscillations $\Delta R_{xx}$ are plotted versus $1/B_{⊥}$ in Figure 4e at the same $V_{G}=-2.55$ V. Assuming a circular Fermi surface, the lowest‐harmonic SdH oscillation observed is periodic in 1/B with period 2πph/e [33], and using this relation, we find a hole concentration $p=1.41\times 10^{11}\;{\mathrm{cm}}^{-2}$, which is very close to that extracted from the Hall effect, as expected for a QW with negligible bulk transport contribution.

**FIGURE 5:**
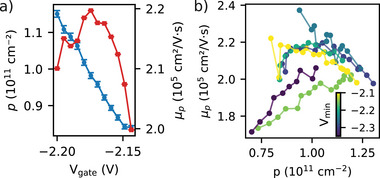
(a) Extracted carrier concentration p (blue curve) and mobility $\mu_{p}$ (red curve) for $V_{\min}=-2.2$ V, as a function of $V_{G}$. (b) Extracted Hall mobility versus extracted Hall carrier concentration for different values of $V_{\min}$ ranging from −2.1 V (yellow) to −2.35 V (blue).

Next, we discuss the electronic hole mobility $\mu_{p}$ and concentration p, measured as a function of gate voltage to assess the quality of the accumulation‐mode quantum well HBs. The gate voltage $V_{G}$ was swept from 0 to $V_{\min}$, and back to 0, for different $V_{\min}$ values. At each $V_{G}$, the magnetic field $B_{⊥}$ was varied to obtain $R_{xx}$ and $R_{xy}$, and the Hall mobility $\mu_{p}$ and hole concentration p were obtained from the relations $R_{xx}=\left(W/L\right)/\left(\mu_{p}pe\right)$ and $R_{xy}=B_{⊥}/\left(ep\right)$. Data for p and $\mu_{p}$ are shown in Figure 5a for $V_{G}$ ranging from −2.14 V to −2.2 V at $V_{\min}=-2.2$ V. The carrier concentration p increases approximately linearly as $V_{G}$ decreases, as expected. Starting from lower hole concentration, the mobility initially increases with increasing p, reaches a maximum value of about $2.2\times 10^{5}\;{\mathrm{cm}}^{2}/\left(V\;s\right)$, and then decreases for further increases in p. The overall upward trend at low concentration is similar to what is reported in natural Ge/${\mathrm{Si}}_{x}{\mathrm{Ge}}_{1-x}$ heterostructures [32], and can be attributed to better screening of Coulomb impurities as carrier concentration increases. Further decrease in gate voltage (i.e., increased carrier concentration) populates positive charge traps at, e.g., the ${\mathrm{Si}}_{x}{\mathrm{Ge}}_{1-x}$/Si/${\mathrm{SiO}}_{2}$ interface, thereby reducing carrier mobility.

Reliable Hall voltages were difficult to extract for gate voltages just below the turn‐on voltage, where the Hall bar resistance $R_{xx}$ varied on a timescale much longer than that required to sweep the magnetic field. We attribute this slow variation of device resistance to slow changes in the population of trapped charges (and therefore hole concentration p) at the semiconductor–dielectric interface, ${\mathrm{Si}}_{x}{\mathrm{Ge}}_{1-x}$/Si/${\mathrm{SiO}}_{2}$/${\mathrm{Al}}_{2}O_{3}$. The change in hole concentration p introduces a slow drift in $R_{xx}$ and $R_{xy}=B_{⊥}/\left(ep\right)$, making the extraction of p and $\mu_{p}$ as a function of $B_{⊥}$ unreliable.

To study the impact of interface charges on the transport properties, we plot the dependence of the extracted Hall mobility $\mu_{p}$ on the extracted carrier concentration p, for different values of $V_{\min}$, during the negative sweep of $V_{G}$ from 0 to $V_{\min}$. Recall that for smaller values of $V_{\min}$, the turn‐on voltage shifts negatively, confirming that trapped charges become more positive. The variation of turn‐on voltage with $V_{\min}$ is plotted in Figure S1a (Supporting Information). The Hall mobility as a function of carrier concentration for different values of $V_{\min}$ is shown in Figure 5b. The relationship between mobility and hole concentration follows a sensible trend for each value of $V_{\min}$. At low hole concentrations, as hole concentration increases, mobility increases, as expected when we move away from the mobility edge (percolation threshold). As hole concentration increases further, the mobility saturates, and eventually starts to decrease, as expected if the increasing population of scattering centers outweighs incremental improvements in mobility due to improved screening of each trapped charge. Notably, while the mobility curves for each value of $V_{\min}$ bunch together at high trap concentrations, the plateau in mobility at intermediate hole concentrations do not follow a systematic trend with $V_{\min}$, possibly because the trapped charge populations do not reach a steady‐state in the experiment.

The percolation concentration $p_{p}$ is an important figure of merit for laterally gated quantum dots formed in quantum wells (QWs). This is because the percolation concentration quantifies the disorder at low carrier densities relevant to, e.g., few‐hole quantum dots. We measured monotonically increasing conductance for carrier concentrations as low as $7\times 10^{10}\;{\mathrm{cm}}^{-2}$, and therefore infer that the percolation concentration lies below this value. A reliable extrapolation of the conductance versus concentration data to zero conductance is, however, difficult with our data (see Figure S1b, Supporting Information).

We now briefly comment on how the properties of the purified material compare to literature values. The peak mobility of $2.4\times 10^{5}\;{\mathrm{cm}}^{2}/\left(V\;s\right)$ at a carrier concentration of $9\times 10^{10}\;{\mathrm{cm}}^{-2}$ and a percolation concentration below $7\times 10^{10}\;{\mathrm{cm}}^{-2}$ indicate high material quality suitable for studying single‐hole coherence in laterally gated quantum dots, in these nuclear spin‐free 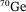/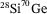 heterostructures. Reported values of mobility (percolation concentration) in the literature range from $7\times 10^{4}$ to $5\times 10^{5}\;{\mathrm{cm}}^{2}/\left(V\;s\right)$ ($4\times 10^{10}$ to $1.2\times 10^{11}\;{\mathrm{cm}}^{-2}$) in undoped QWs with barriers of comparable composition at temperatures around 1 K [34, 35, 36, 37, 38, 39]. Representative literature values are summarized in Table S2 (Supporting Information). Generally, higher mobilities (lower percolation concentrations) are expected for larger QW depths, as this brings the QW further from the ${\mathrm{Si}}_{x}{\mathrm{Ge}}_{1-x}$/Si/${\mathrm{SiO}}_{2}$/${\mathrm{Al}}_{2}O_{3}$ interface.

It is worth noting that the above comparisons are limited due to small differences in the heterostructure layer thicknesses, barrier compositions, and related strain. Nevertheless, some additional observations are instructive. A trap population independent of gate voltage (carrier concentration) would likely yield a mobility that increases with carrier concentration due to improved screening of defects. However, this stands in contrast to our observations, as the mobility consistently plateaus and then decreases with increasing carrier concentration, regardless of $V_{\min}$. We therefore speculate that filling of interface traps via the gate, which increases as $V_{G}$ decreases (carrier concentration increases), produces a scattering potential that limits the mean free path at high carrier concentrations. It is thus possible, at least for the highest hole concentrations reported, that the parameters measured here for HBs are limited by traps at an interface formed during device processing, rather than by the intrinsic heterostructure interfaces.

## 4 Conclusion

In summary, to prevent cross‐contamination from natural precursors and minimize the use of isotopically enriched gases without compromising crystalline quality, this work demonstrates the epitaxial growth of nuclear spin‐free 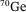/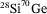 heterostructures on industrial SiGe buffers using reduced‐pressure chemical vapor deposition with highly purified 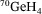 (>99.9%) and 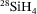 (>99.99%). The resulting heterostructures exhibit a dislocation density of $5.3\times 10^{6}\;{\mathrm{cm}}^{-2}$ and an isotopic purity above 99.99%, free of carbon and oxygen impurities, as confirmed by atom probe tomography. Hall bar measurements show precise gate control of hole density and high mobilities ($∼2.4\times 10^{5}\;{\mathrm{cm}}^{2}/V\;s$), with transport limited by interface traps and percolation below $7\times 10^{10}\;{\mathrm{cm}}^{-2}$. These results establish high‐purity 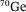/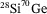 heterostructures as a promising platform for long‐coherence hole spin qubits, free from hyperfine interactions with 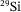 and 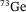 nuclei.

## 5 Methods

*Surface Cleaning and Epitaxial Growth*. Before the overgrowth, the industrial buffers were cleaned in 2 wt% HF and 25 wt% HCl while omitting the final deionized (DI) rinse. As a final step, the buffers were in situ annealed in hydrogen at 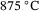 for 30 min. The growth was performed in a thermal reduced‐pressure chemical vapor deposition (RPCVD) system using hydrogen as a carrier gas and centrifugally enriched monogermane (12% 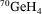 in $H_{2}$ with isotopic purity >99.9%) and monosilane (25% 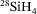 in $H_{2}$ with isotopic purity >99.99%). The total growth pressure was 20 Torr, with partial pressures of ∼2–9 Pa for 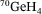 and ∼6 Pa for 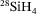. The growth temperature was optimized at 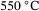 following tests in the 600–550 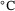 range to minimize the formation of Ge islands at the start of regrowth and to promote sharp quantum well (QW) interfaces.

When initiating the homoepitaxy, an initial boost in 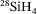 flow for a few seconds was required to further suppress Ge island formation. When the desired first barrier growth time was reached, the precursor flows were cut for approximately 30 s, during which the reactor was purged with a 2700 sccm hydrogen flow. The growth of the 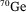 well then followed, succeeded by a 30 s hydrogen purge of the reactor. To ensure a sharp QW/second‐barrier interface, a second boost in the 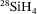 precursor flow was performed to correct the silicon concentration profile. Finally, a ∼2 nm capping layer of 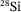 was grown to protect the purified heterostructure with a stable native oxide.

*Characterization*. Thin specimens suitable for cross‐sectional transmission electron microscopy (XTEM) were prepared using a focused ion beam (FIB) in a FEI Helios NanoLab 600 operating with a 30 keV Ga ion beam. TEM imaging was performed on a C‐FEG JEOL JEM‐Fx2000 operating at 200 kV. To establish the isotopic purity of the material and quantify possible contaminants, laser‐assisted atom probe tomography (APT) measurements were conducted using an Invizo 6000 instrument with a picosecond laser at a wavelength of 257 nm, pulse energy of 25–45 pJ, and base temperature of 25 K. Monte‐Carlo analyses of the time‐of‐flight mass spectra are utilized to establish the average and standard deviations on the isotope contents. Graph data points are established for 2 nm layers, corresponding to a total pool of millions of atoms over 3‐4 APT tips per sample. The methodology is described in more detail in the SI of ref [26]. Surface roughness of the starting buffers and the 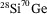 overgrowth was analyzed using a Bruker Icon FastScan atomic force microscope (AFM) in PeakForce quantitative nanomechanics (QNM) tapping mode over 10–20 μm scan areas. Surface profiles were corrected in Bruker Nanoscope software using a second‐order plane fit. Threading dislocation densities in the heterostructures and commercial buffers were estimated using defect delineation experiments based on a Secco etch (1 part HF, 49 wt%, to 2 parts 0.15 mol/L $K_{2}{\mathrm{Cr}}_{2}O_{7}$). Pits were etched at room temperature for 20–60 s and analyzed using AFM. Crystallinity of the QW was evaluated by high‐resolution X‐ray diffraction (XRD) using a Bruker D8 Discover system equipped with a Cu $K\alpha_{1}$ source, a triple‐bounce Ge(220) analyzer, and a Ge(220) monochromator. The ($\bar{2}\;\bar{2}\;4$) reflection was used for reciprocal space mapping (RSM) analysis.

*Device Processing*. The devices feature titanium/gold bilayer bond pads, aluminum ohmic contacts, an ${\mathrm{Al}}_{2}O_{3}$ gate dielectric deposited on the Si/${\mathrm{SiO}}_{2}$ cap, and a titanium/gold bilayer top gate. A silicon nitride field dielectric was selectively deposited such that it appears only below the gate bond pad in the finished device. The ${\mathrm{Al}}_{2}O_{3}$ gate dielectric was deposited after the silicon nitride. The QW region was defined by selective‐area reactive ion etching (RIE) to remove the quantum well around the Hall bar (HB) boundary. The mesa etch employed an ${\mathrm{SF}}_{6}$/$O_{2}$ chemistry and removed approximately 100 nm of material, including the QW.

The Al ohmic contacts were deposited by the lift‐off technique employing electron‐beam evaporation through a polymer resist mask, on top of the Si‐capped Ge heterostructure, without a via to the quantum well. Al deposition was carried out after a 15 s dip in buffered hydrofluoric acid employed to remove the native ${\mathrm{SiO}}_{2}$ layer on the Si cap, without a via to the Ge quantum well. Following the Al ohmic contact deposition, the ${\mathrm{Al}}_{2}O_{3}$ gate dielectric (22 nm thick) was deposited by thermal atomic layer deposition (ALD) at 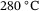. The total time spent at 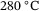 during the ALD processing step was around 60 minutes, which is expected to alloy/dope Al partially into the heterostructure, bringing the Al metal into electrical contact with the buried QW. The silicon nitride (≈250 nm thick) was deposited by pulsed DC reactive sputtering at room temperature to enhance the electrical robustness of the gate bond pad for wire bonding. Aluminum ohmic contacts were annealed in situ during the deposition of the ${\mathrm{Al}}_{2}O_{3}$ gate dielectric. A schematic cross‐section of the device is shown in Figure 3b.

*Transport Measurements*. Hall bar measurements were performed in a cryo‐free variable temperature insert (VTI) at approximately 1.4 K, in the bore of an 8 T superconducting solenoid. The current I between the outer contacts was measured as a function of $V_{G}$ by applying an oscillating voltage $V_{\mathrm{bias}}=50\;\mu V$ to one contact and recording the resulting current at the opposite contact using a Stanford SR860 lock‐in amplifier.

For magneto‐transport measurements, longitudinal ($R_{xx}=V_{xx}/I$) and transverse ($R_{xy}=V_{xy}/I$) resistances were measured by applying an oscillating bias voltage $V_{\mathrm{bias}}=40\;\mathrm{mV}$ across a series resistor $R_{s}=4\;M\Omega \gg R_{xx}$, giving $I=V_{\mathrm{bias}}/R_{s}=10\;\mathrm{nA}$. Voltages $V_{xx}$ and $V_{xy}$ were measured differentially using SR860 lock‐in amplifiers. The gate voltage $V_{G}$ and temperature were held constant while sweeping the perpendicular magnetic field $B_{⊥}$. The gate voltage was swept from 0 to $V_{\min}$, then from $V_{\min}$ back to 0, for progressively more negative $V_{\min}$ values to probe gate‐induced charging of interface traps. When necessary, devices were reset by cycling the VTI temperature to 300 K to release trapped charges that shifted the turn‐on voltage.

## Conflicts of Interest

The authors declare no conflicts of interest.

## Supporting information


**Supporting File**: advs74221‐sup‐0001‐SuppMat.pdf.

## Data Availability

The data that support the findings of this study are available from the corresponding author upon reasonable request.
