# Supplementary material for: Nuclear Spin‐Free 70Ge/28Si70Ge Quantum Well Heterostructures Grown on Industrial SiGe‐Buffered Wafers
Source: Adv Sci (Weinh). 2026 Feb 16;13(21):e23504. doi: 10.1002/advs.202523504 (PMC13073280; doi:10.1002/advs.202523504)
Supplement: Supplementary file 1 — Supporting File: advs74221‐sup‐0001‐SuppMat.pdf. [file ADVS-13-e23504-s001.pdf]

Nuclear spin-free $^{70}Ge/^{28}Si^{70}Ge$ quantum well heterostructures grown on industrial SiGe-buffered wafers

Supplementary Material

P. Daoust,[1,] N. Rotaru,[1] S. Koelling,[1] É. Rahier,[1] A. Dubé-Valade,[1] P. Del Vecchio,[1]
D. Biswas,[2,3] M. S. Edwards,[2,4] M. Tanvir,[2,4] E. Sajadi,[2,4] J. Salfi,[2,4,3] and O.Moutanabbir[1]

[1)] *Department of Engineering Physics, École Polytechnique de Montréal, Montréal, C.P. 6079, Succ. Centre-Ville, Montréal, Québec, Canada H3C 3A7*

[2)] *Stewart Blusson Quantum Matter Institute, University of British Columbia, Vancouver, BC, Canada.*

[3)] *Department of Physics and Astronomy, University of British Columbia, Vancouver, BC, Canada.*

[4)] *Department of Electrical and Computer Engineering, University of British Columbia, Vancouver, BC, Canada.*

Contents

S1. Surface preparation protocol of the industrial SiGe wafers 2
S2. Additional details on the transport measurements 3
S3. AFM and Secco etch experiments details 5
S4. Additional details on the APT results 8
References 9

S1. Surface preparation protocol of the industrial SiGe wafers

TABLE SI: Description of the surface treatment before the overgrowth on $Si_{0.18}Ge_{0.82}$ buffers.

Step	Description
1	1 min dip in 2.5 w% HF
2	5 s rinse in DI water
3	1 min dip in 25 w% HCl
4	5 s rinse in DI water
5	1 min dip in 2.5 w% HF with final nitrogen dry only.
6	Hydrogen anneal at 875 ◦C and 10 torr for 30 min.

A multi-step cleaning process involving diluted HF and HCl cleans, as well as *in situ* hydrogen anneals at high temperature, is performed before the CVD growths. The optimized surface preparation protocol is summarized in Table SI and calls for successive cleans in HF and HCl before a final in situ hydrogen anneal.

It was found that germanium-rich $Si_{0.18}Ge_{0.82}$ buffers are not passivated by typical diluted HF dips normally employed in the surface preparation of silicon surfaces. Indeed, the surface remains strongly hydrophilic following even repeated 2-minute dips in 2.5 wt% HF. As hydrophilicity is thought to be an indication of residual surface oxides that can be detrimental to epitaxial growth, additional cleaning steps were introduced.

Following Ponath *et al.*[1], a 25 wt% HCl solution was employed to remove potential germanium suboxides. The surface is completely hydrophobic following this dip. In addition, the surface passivation of these substrates is sensitive to contact with DI water. After a 5-minute rinse following the chemical clean, the buffer is found to be hydrophilic again, and exposure to aqueous media should be minimized. Anneals at high temperature and in a hydrogen atmosphere were found to be essential to obtain good quality material in the case of $Si_{0.18}Ge_{0.82}$ buffers, as indicated by XRD measurements and the mirror finish of the samples.

S2. Additional details on the transport measurements

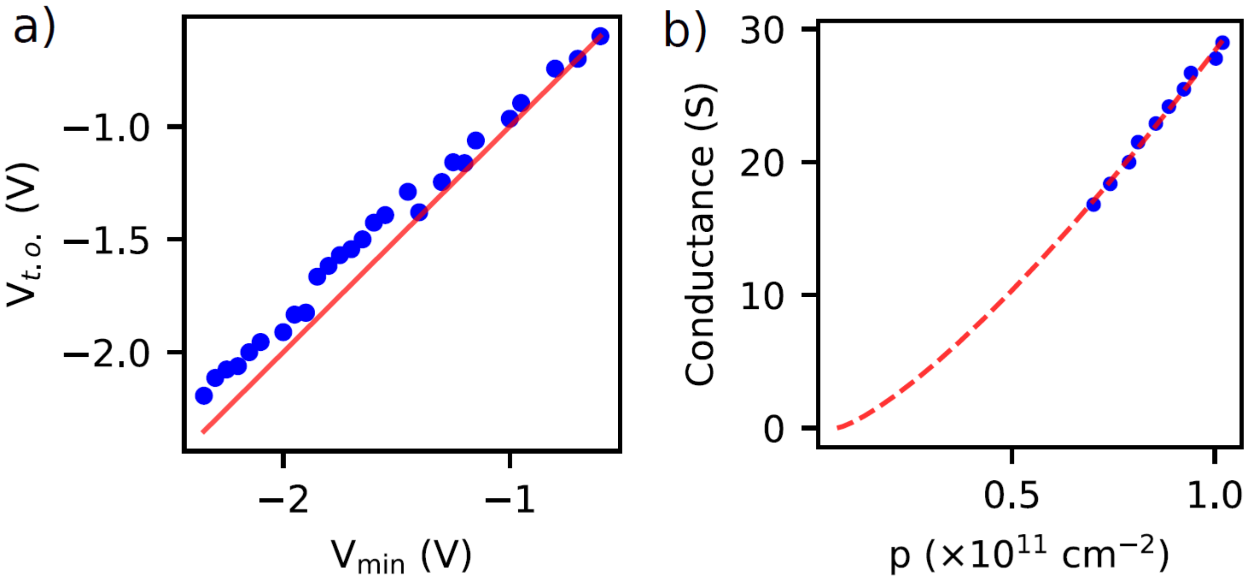

FIG. S1: (a) The turn on voltage is plotted for each V_{min}. The solid red line is the y=x line. (b) Conductance vs carrier concentration for $V_{min} = -2.35$V is shown. The dashed red line is a fit using 2DHG model for percolation concentration.

The turn-on voltage is defined as the highest voltage value corresponding to a V_{min} sweep where the conductance is higher than the conductance at $V_G = 0$. The $V_{t.o.}$ is roughly linear up to the $V_{min} = -2.35$ V, showing no sign of saturation of interface traps. As seen from FIG. S1(a), the first time the device turns on is at $V_G = -0.6$ V. The turn-on voltage shifts with respect to the minimum gate voltage applied with a slope slightly less than unity. A slope of precisely unity would imply that all charge induced by the gate is stored in trapped charge that shifts the gate threshold on experimental timescales. A slope slightly less than unity, like the slope we observe, implies that a very significant fraction of the charge induced by the gate is trapped at the interface. Nevertheless, because the quantum well is relatively deep, the carrier transport properties such as mobility and percolation density are close to the state-of-the-art.

A conductance vs carrier concentration graph is shown in FIG. S1(b). The lowest carrier concentration value where we extracted a Hall signal was $0.7\times10^{11}\text{cm}^{-2}$. This plot confirms that the percolation concentration is lower than $0.7 \times 10^{11}\text{cm}^{-2}$, but to get a more accurate value, we would need data at lower carrier concentrations.

Table S2 below provides an overview of transport characterization results found in the literature for comparable QW structures.

TABLE S2: Summary of reported parameters for strained $Si_{0.2}Ge_{0.8}$/Ge quantum wells.

Reference	Depth	Width	Temp.	Mass	Peak mobility	Min conc.	Max conc.	Percolation conc.	Quantum lifetime
-	nm	nm	K	m_0	cm^2/Vs	cm^{-2}	cm^{-2}	cm^{-2}	ps
Sammak et al.[2]	22	16	1.7	0.090	5.0×10^5	1.5×10^{11}	6.0×10^{11}	1.2×10^{11}	0.74
Massai et al.[3]	47	20	0.015	–	1.5×10^6	3.0×10^{10}	2.2×10^{11}	1.5×10^{10}	–
Li et al.[4]	33.6	8.6	1.7	0.103	5.2×10^5	1.2×10^{11}	5.2×10^{11}	6.9×10^{10}	1.2–2.3
Zhang et al.[5]	35	18	1.5	0.079	9.0×10^4	8.0×10^{10}	2.0×10^{11}	3.7×10^{10}	0.5
Sangwan et al.[6]	20	15	1	–	7.0×10^4	5.0×10^{10}	3.0×10^{11}	2.3×10^{10}	–
Kong et al.[7]	32	16	0.016	0.072	2.0×10^6	7.0×10^{10}	2.0×10^{11}	5.6×10^{10}	1.7
This work	55	20	1.38	-	2.4×10^5	7.0×10^{10}	1.3×10^{11}	less than 7×10^{10}	-

S3. AFM and Secco etch experiments details

To investigate the state of the surface at various points in the sample fabrication, AFM measurements were done in QNM mode before and after growth. Table S3 summarises these different measurements. A representative AFM map for the grown heterostructure surface, showing the typical crosshatch pattern, is presented in FIG. S2.

TABLE S3: Summary of AFM measurements at different stages of sample processing.

	Arithmetic roughness R_a (nm)	RMS roughness R_q (nm)	Sampled area Height range Z (nm)
Initial $Si_{0.18}Ge_{0.82}$ Buffer	1.5	2.0	13.2
Post chemical clean	2.1	2.7	20.0
Post growth	2.0	2.5	14

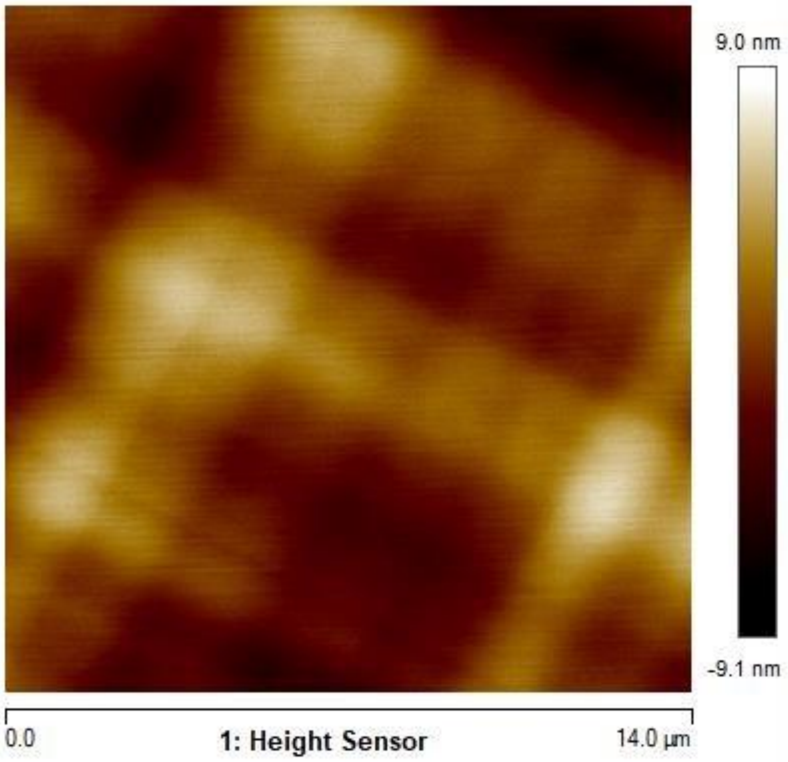

FIG. S2: Typical AFM height map obtained for the optimized QW growths of this work.

To estimate the density of threading dislocation defects in the industrial buffers and for the grown heterostructures, standard Secco etches[8] were performed over several etch times (20s-60s). FIGs S3 and S4 present AFM map resolved defects associated with threading dislocations. For both series of results, the defects grow in size over time, strongly implying that they are being revealed by the Secco solution. The obtained average density of defects is calculated to be $7.8 \cdot 10^6$ cm^{-2} for the industrial buffers and $5.3 \cdot 10^6$ cm^{-2} for the grown QW heterostructures, *i.e.,* they are of the same order of magnitude.

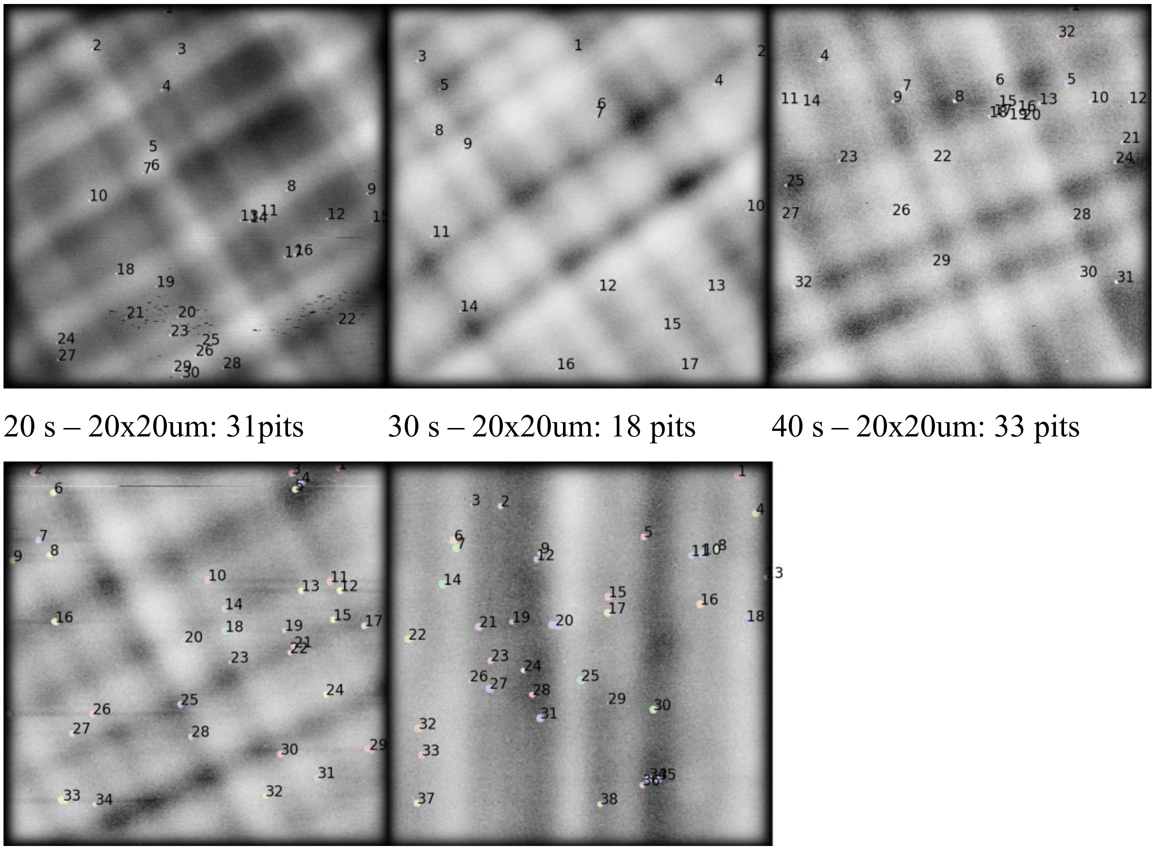

20 s – 20x20um: 31pits   30 s – 20x20um: 18 pits   40 s – 20x20um: 33 pits

50 s – 20x20um: 35 pits   60 s – 20x20um: 39 pits

FIG. S3: AFM maps following Secco etches with different etch times ($Si_{0.18}Ge_{0.82}$ buffers).

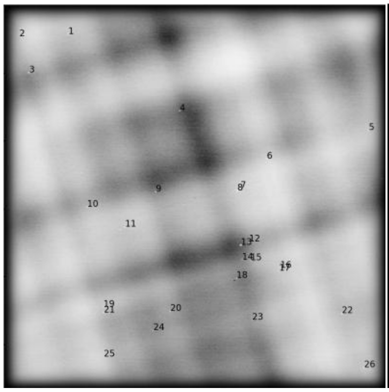

20 s – 20x20um: 27pits

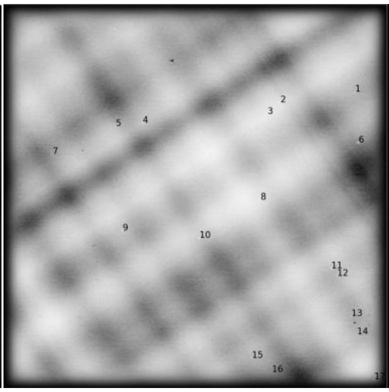

30 s – 20x20um: 18 pits

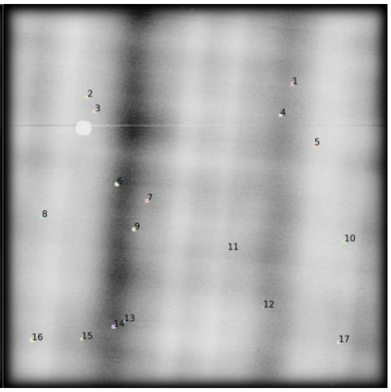

40 s – 20x20um: 18 pits

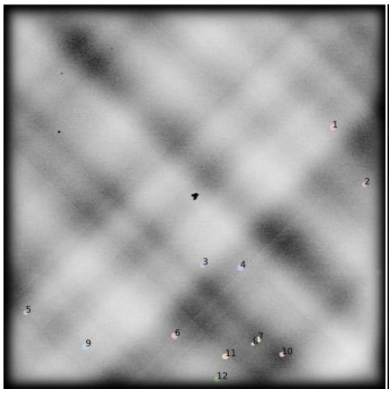

50 s – 20x20um: 13 pits

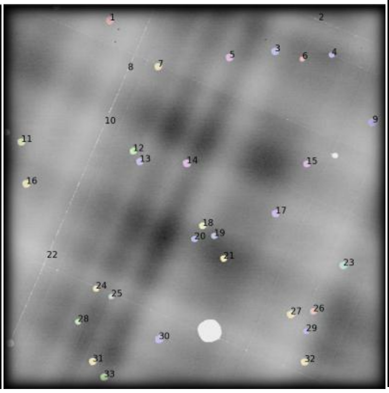

60 s – 20x20um: 34 pits

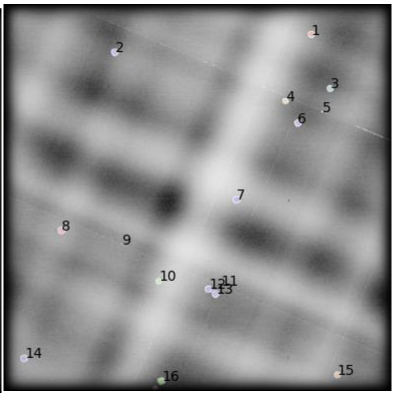

60 s – 20x20um: 17 pits

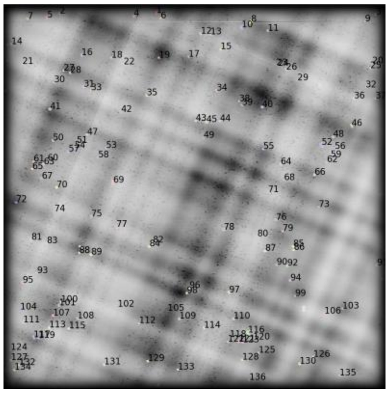

60 s in 50x50 um: 137 pits

FIG. S4: AFM maps following Secco etches with different etch times (overgrown heterostructures).

S4. Additional details on the APT results

For initial unoptimized samples, APT of the buffer/heterostructure growth interface reveals Ge-rich areas with widths of ~100 nm and heights of ~20-30 nm (FIG. S5). The AFM maps of these first samples show features of roughly similar dimensions and high roughness in the tens of nm, implying that the shape of the Ge-rich areas propagates during the growth. In order to mostly eliminate this issue, the growth temperatures were lowered from 600°C to 550°C, and the partial pressure of $^{28}SiH_4$ was increased for a few seconds when initiating the growth. This is believed to favor the growth of SiGe and deter the 3d growth of Ge.

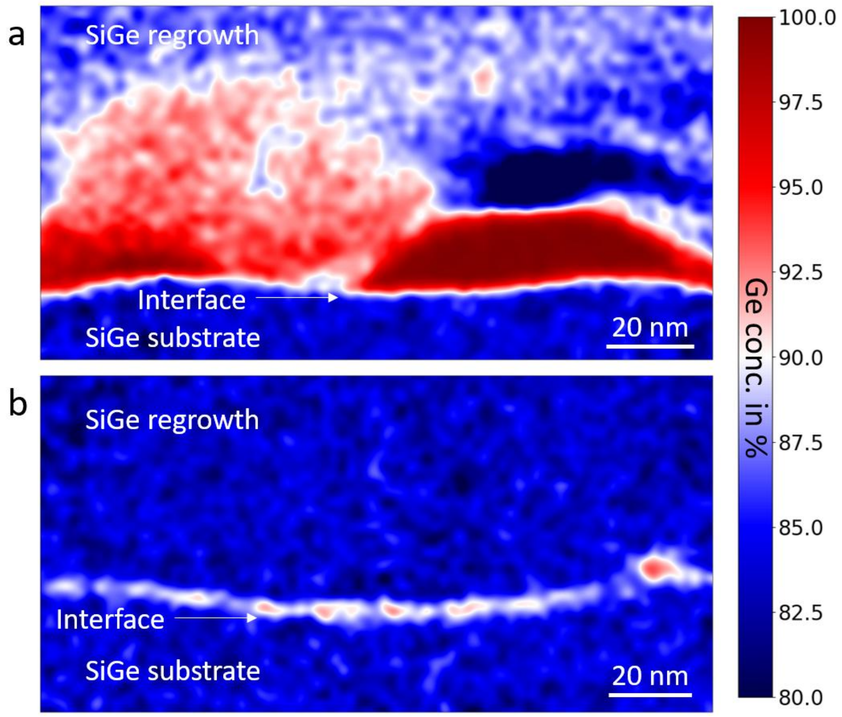

FIG. S5: Ge concentration at the SiGe regrowth interface as measured by APT before (a) and after (b) optimization of the regrowth. The images show the Ge concentration in a 3nm thick slice cut perpendicular to the interface.

References

1. P. Ponath, A. B. Posadas, and A. A. Demkov, Ge(001) surface cleaning methods fordevice integration, *Applied Physics Reviews*, vol. 4, p. 021308, 06 2017.
2. A. Sammak, D. Sabbagh, N. W. Hendrickx, M. Lodari, B. Paquelet Wuetz, A. Tosato, L. Yeoh, M. Bollani, M. Virgilio, M. A. Schubert, P. Zaumseil, G. Capellini, M. Veldhorst, G. Scappucci, *Adv. Funct. Mater.* 2019, 29, 1807613.
3. Massai, L., Hetényi, B., Mergenthaler, M. *et al.* Impact of interface traps on charge noise and low-density transport properties in Ge/SiGe heterostructures. *Commun Mater* **5**, 151 (2024).
4. Li Y X, Kong Z Z, Hou S M, et al. Quantum transport quality of a processed undoped Ge/SiGe heterostructure. Phys Rev B, 2023, 108, 045303.
5. Jie-yin Zhang, Ming Ming, Jian-huan Wang, Ding-ming Huang, Han Gao, Yi-xin Chu, Bin-xiao Fu, H. Q. Xu, Jian-jun Zhang; High-quality Ge/SiGe heterostructure with atomically sharp interface grown by molecular beam epitaxy. *Appl. Phys. Lett.* 16 September 2024; 125 (12): 122106.
6. N. Sangwan, E. Jutzi, C. Olsen, S. Vogel, A. Nigro, I. Zardo, and A. Hofmann, Impact of surface treatments on the transport properties of germanium 2DHGS, arXiv preprint arXiv:2411.03995 (2024).
7. Z. Kong, Z. Li, G. Cao, J. Su, Y. Zhang, J. Liu, J. Liu, Y. Ren, H. Li, L. Wei, et al., Undoped strained ge quantum well with ultrahigh mobility of two million, ACS Applied Materials & Interfaces 15, 28799 (2023).
8. F. Secco d' Aragona, Dislocation etch for (100) planes in silicon, *Journal of The Elec-trochemical Society*, vol. 119, p. 948, jul 1972.
